# Relational and Growth Outcomes Following Couples Therapy With MDMA for PTSD

**DOI:** 10.3389/fpsyt.2021.702838

**Published:** 2021-06-28

**Authors:** Anne C. Wagner, Rachel E. Liebman, Ann T. Mithoefer, Michael C. Mithoefer, Candice M. Monson

**Affiliations:** ^1^Remedy, Toronto, ON, Canada; ^2^Department of Psychology, Ryerson University, Toronto, ON, Canada; ^3^Department of Psychiatry, Toronto General Hospital, University Health Network, Toronto, ON, Canada; ^4^MAPS Public Benefit Corporation, Santa Cruz, CA, United States

**Keywords:** MDMA, PTSD, couples, treatment, post-traumatic growth, interpersonal functioning

## Abstract

Healing from trauma occurs in a relational context, and the impacts of traumatic experiences that result in post-traumatic stress disorder (PTSD) go beyond the diagnosis itself. To fully understand a treatment for PTSD, understanding its impact on interpersonal, relational, and growth outcomes yields a more fulsome picture of the effects of the treatment. The current paper examines these secondary outcomes of a pilot trial of Cognitive Behavioral Conjoint Therapy (CBCT) for PTSD with MDMA. Six romantic dyads, where one partner had PTSD, undertook a course of treatment combining CBCT for PTSD with two MDMA psychotherapy sessions. Outcomes were assessed at mid-treatment, post-treatment, and 3- and 6-month follow-up. Both partners reported improvements in post-traumatic growth, relational support, and social intimacy. Partners reported reduced behavioral accommodation and conflict in the relationship, and patients with PTSD reported improved psychosocial functioning and empathic concern. These improvements were maintained throughout the follow-up period. These findings suggest that CBCT for PTSD with MDMA has significant effect on relational and growth outcomes in this pilot sample. Improvements in these domains is central to a holistic recovery from traumatic experiences, and lends support to the utility of treating PTSD dyadically.

## Introduction

Traumatic events impact relationships, and healing from trauma occurs in a relational context. Social factors play a key role in the development, worsening, and improvement of post-traumatic stress disorder (PTSD) (1). Negative social interactions in particular are pernicious in their impact on traumatized individuals, and are associated with higher likelihood of PTSD following a traumatic event (1, 2). Likewise, the presence of PTSD can have a deleterious effect on relationships (3), therefore creating a self-perpetuating cycle of exacerbation of symptoms (4). Given the role of social interactions in the course of, and healing from, PTSD, we examined the relational and growth outcomes in a pilot trial of a dyadic treatment for PTSD, Cognitive Behavioral Conjoint Therapy (CBCT) (5) for PTSD with MDMA.

Interpersonal functioning, both within and outside of intimate relationships, is often negatively impacted by PTSD, and vice versa. Addressing this impact is important given the fundamental role interpersonal functioning plays in developing and maintaining relationships over time, attachment, parenting, reducing secondary traumatization, and satisfaction and security in life [e.g., (6, 7)]. Interpersonal traumas more frequently result in PTSD than other forms of trauma in part because of the violation in expectations of relational trust, which then generalizes to other interpersonal connections (8). Additionally, the content of much trauma-related suffering is interpersonal (including feelings of numbness and detachment from others, cognitive appraisals of trust, control, and intimacy, avoidance of people and situations, anger, and aggression as examples). PTSD can erode relationship quality over time, and conversely, relationships can exacerbate or ameliorate the post-trauma recovery, depending on whether the interactions are positive or negative (1).

Post-traumatic growth, a construct consisting of relations to others, perceptions of new possibilities in life, perceived personal strength, spiritual change, and an appreciation of life (9), exists separately from traumatic symptomatology (10). It is an important outcome due to its reflection of an adaptive cognitive process of adjustment (11), and is positively associated with resiliency, meaning-making of the traumatic event, relationship functioning and hope (9, 12). Therefore, post-traumatic growth is indicative of a holistic process of change following a traumatic event.

The loved ones of individuals with PTSD often experience their own struggles in relation to being in relationship with someone who has experienced a significant traumatic event(s) [e.g., (3)]. This can encompass their own mental health and well-being, quality of life, and their interpersonal relationships and relationship satisfaction with the person with PTSD. While well-meaning, loved ones of individuals with PTSD may engage in behavioral accommodation, a means of trying to ease the symptoms of the person with PTSD (e.g., by reducing noise, limiting the responsibilities of the person with PTSD, excusing aggressive behavior, etc.), which inadvertently may reinforce the presence of these symptoms (13). Overcoming PTSD ideally occurs in tandem with a greater engagement in life. To foster and support holistic healing, domains related to interpersonal functioning, quality of relationships, and post-traumatic growth need to be considered, as opposed to solely focusing on psychological symptomatology.

Treating PTSD in a relational framework, and in this case, a couple format, is one means of explicitly utilizing the interpersonal milieu in order to create substantive and lasting change, for both the person with PTSD and their loved one. Cognitive Behavioral Conjoint Therapy for PTSD (5), a dyadic intervention for PTSD, has shown significant positive impact on both patient and partner mental health and well-being (14, 15), as well as relational functioning and post-traumatic growth (16).

3-4 methylenedioxymethamphetamine (MDMA) has been used in couple therapy since its first clinical applications in the 1970s (17–19). MDMA's empathogenic qualities create a unique therapeutic opportunity in which couples not only feel more comfortable sharing their emotions, but can also approach conversations with greater ease (20). MDMA has been reported to facilitate individuals' ability to maintain an optimal window of tolerance, allowing for both positive and negative emotions to occur without avoidance or being overwhelmed (21). This can be particularly useful in addressing couple-based distress and communication.

MDMA-assisted psychotherapy for PTSD has shown promising results as an individual treatment for PTSD in an inner-directed, supportive therapeutic framework [e.g., (22)]. Participants also report improvements in psychosocial functioning and interpersonal relationships following the treatment, albeit without the use of standardized measures (23), as well as in post-traumatic growth (24). Collateral reports of symptoms and outcomes by close others have not yet been collected in MDMA-assisted individual psychotherapy studies, offering an opportunity to expand the understanding of the impacts of the intervention.

We therefore sought to examine patient and partner outcomes related to interpersonal functioning, relationship satisfaction, and post-traumatic growth in a sample of dyads who participated in a pilot study of Cognitive Behavioral Conjoint Therapy for PTSD with MDMA (25). Primary outcomes from this trial demonstrated significant decreases in PTSD symptoms assessed by independent rater (*d* = 2.10), the individual with PTSD (*d* = 2.72), and their partner (*d* = 1.85) (25). Treatment gains continued to improve by 6-month follow-up (*d* = 2.25, 3.59, 2.72, respectively). The combination of CBCT for PTSD and MDMA was designed to amplify the qualities of each, and in particular, capitalize upon the relational context each is designed for.

## Method

### Participants

Six dyads participated in the uncontrolled pilot trial, which was conducted in a private practice clinic in Charleston, South Carolina. One member of each dyad had PTSD (henceforth referred to as the patient), while the partner did not. Inclusion criteria for both partners included being 18 or older, no current substance use disorder, no active suicidal planning or intent, mania, psychosis, or severe partner aggression. Participants were required to taper off all psychiatric medications, and were medically screened for contraindicated conditions, including essential hypertension, cardiac disorder, or any other major medical condition.

For all participants, the average age was ~47 years, all were White, and they were all in mixed gender partnerships. For the participants with PTSD, two were female, all had co-morbid psychological diagnoses, and all had received prior psychological and pharmacological PTSD treatments. They had experienced a range of traumatic events, with five of the six having experienced multiple traumatic events (including childhood physical abuse, childhood sexual abuse, and combat). All of the partners were White, four were female, and 50% had psychological diagnoses.

Ethics approval was received from Ryerson University and the WCGIRB. All participants provided complete and ongoing informed consent. For a full study description, see Monson et al. (25).

### Measures

Participants completed assessments at pre-treatment, mid-treatment, post-treatment, and 3- and 6-month follow-up. The following measures were used:

#### Relationship Aggression Outcomes

The Revised Conflict Tactics Scale (CTS-2) (26) contains 39 methods of managing conflict, answered for both having used and having experienced from their partner. The measure comprises five subscales (rated from 0 to 6): Negotiation (subdivided into emotional and cognitive negotiation strategies, considered positive behaviors with statements such as “I showed respect for my partner's feelings about an issue” and “My partner suggested a compromise to a disagreement”); Psychological aggression; Physical assault; Sexual coercion; and Physical injury. The scale has demonstrated good internal consistency (subscale α = 0.75–0.95) (26). Presence of severe aggression in the sexual coercion or physical aggression subscales were exclusionary criteria at baseline, and therefore baseline levels of aggression in the sample were low. Both members of the couple completed the CTS-2. Severe relationship aggression (as assessed by the CTS-2) was a rule-out for study inclusion. In order to establish that the treatment did not worsen aggression over time, we examined changes in minor aggression.

#### Trauma-Related Outcomes

The Post-Traumatic Growth Inventory (PTGI) (9) was completed by both members of the dyad, with each referencing post-traumatic growth in the partner with PTSD. The PTGI is a 21-item self-report measure of perceived growth. It was edited to be collateral-report for the purpose of this study. Each item is scored on a 6-point scale from “I did not experience this change as a result of my crisis” to “I experienced this change to a very great degree as a result of my crisis.” The scale has demonstrated good internal consistency across studies [e.g., (9, 27)].

The Significant Others' Responses to Trauma Scale (SORTS) (13) is a 14-item measure completed only by the partner of the person with PTSD. It assesses relationship problems and distress associated with behaviors meant to accommodate PTSD symptoms. Each item is rated for both frequency and intensity on a five-point Likert scale (from 0 to 4). Example items include “How much have you canceled or rearranged plans or social activities because (trauma survivor) did not want to do them?” and “Have you had to take over a task or chore for (trauma survivor) that he/she is uncomfortable doing because of his/her traumatic event?.” The SORTS has demonstrated strong internal consistency (α = 0.93 for total score).

#### Relationship Quality Outcomes

Quality of Relationships Inventory (QRI) (28) is a 25-item self-report measure that assesses perceived support from a chosen relationship, the amount of conflict within this relationship, and the perception of the relationship as deep and secure. The QRI consists of three subscales: Support, Conflict, and Depth. Items are rated on a 4-point Likert scale from “Not at all” to “Very much.” Example questions include “To what extent could you turn to this person for advice about problems?” and “How critical of you is this person?.” The QRI has demonstrated good to excellent internal consistency across studies and type of relationship (α = 0.7–0.9) (28, 29).

#### Psychosocial Functioning

Inventory of Psychosocial Functioning (IPF) (30) is an 80-item self-report measure developed to assess functioning across domains of romantic relationships, family, work, friendships, parenting, education, and self-care. Responses are rated on a 7-point Likert-scale from 0 “never” to 6 “always.” Higher scores indicate greater functional impairment. Example questions include “I had trouble settling arguments or disagreements with my spouse or partner” and “I had trouble showing up for work on time.” The measure has demonstrated strong internal consistency (e.g., α = 0.79–0.92) (30).

Miller Social Intimacy Scale (MSIS) (31) is a 17-item self-report measure designed to assess current social intimacy experienced across relationships. The scale contains six items assessing frequency of socially intimate feelings and behaviors and 11 items assessing depth of social intimacy. Example items include “When you have leisure time how often do you choose to spend it with him/her alone?” and “How close do you feel to him/her most of the time?.” All items are rated on a 10-point Likert scale, with the total score as a single factor. The measure has demonstrated strong internal consistency (e.g., α = 0.84–0.95) (31, 32).

Interpersonal Reactivity Index (IRI) (33) is a 28-item self-report scale assessing empathic tendencies. It consists of four subscales: Perspective taking (the ability to adopt another's perspective); Fantasy (the tendency to identify with fantasies); Empathic Concern (the tendency to experience concern, warmth, and sympathy toward others); and Personal Distress (the tendency to experience distress when witnessing others' negative experiences) (34). Items are measured on a 5-point, Likert-type scale. This measure has demonstrated good internal consistency (α = 0.71–0.77) (33).

### Procedure

Participants were treated by a co-therapy team in a course of CBCT for PTSD with the addition of 2 full-day MDMA sessions. CBCT for PTSD typically consists of 15 protocolized sessions. For this intervention, the protocol was delivered over a total of 7 weeks, with five modules of CBCT delivered (in 1.5 days) prior to MDMA session one, six modules of CBCT (delivered biweekly and then two the day before the second MDMA session) between MDMA sessions one and two, and the remaining four modules of CBCT (delivered weekly) following MDMA session two.

CBCT for PTSD consists of three phases – the first phase highlights safety-building including psychoeducation about traumatic reactions and disclosure of traumatic events, along with tools for managing anger. The second phase focuses on the development of shared communication skills and begins the process of reducing avoidance, a key contributor in the maintenance of PTSD symptoms, by having the dyad participate in behavioral approach activities. The third phase focuses on cognitive work to address meaning-making of the trauma and reduce negative cognitive patterns related to both trauma and relational beliefs. Both members of the dyad engage in all components of the therapy, including the MDMA sessions. By placing equal emphasis on both individual and relationship-level problems, the goal is to heal PTSD and the relational context in which it exists.

Capitalizing on the empathogenic qualities of MDMA, the MDMA sessions were placed strategically in sections of the CBCT protocol we wanted to amplify, namely immediately following the introduction of communication skills, and in the middle of trauma processing.

MDMA sessions consisted of the administration of 75 mg of MDMA during the first session, with an optional supplemental half-dose of 37.5 mg at 1.5 h after initial administration. During the second MDMA session, the base dose was increased to 100 mg of MDMA, with an optional supplemental half-dose of 50 mg at 1.5 h. During the MDMA sessions, participants were in reclining armchairs with eyeshades and headphones available. Instrumental music was played. Participants were encouraged to alternate time spent “inside,” focusing on internal experiences, and time spent in conversation or sharing with the therapists and their partner. A full description of the procedures and a case example can be found in Wagner et al. (20).

### Data Analyses

All outcomes had a maximum of 1–2 (maximum 30%) missing data points at any given assessment interval.

Analyses were conducted in SPSS Version 26 (35). Growth curve models were used to analyze outcomes at each assessment, with time transformed to be the natural log function of the number of days since baseline. To accurately specify the variance structure of the growth factors, we compared models with fixed and random variance components. A chi-square difference test was conducted to compare the nested models using the log-likelihood based goodness of fit statistic (referred to as the deviance statistic) of the more saturated model (the random effects model) to the less saturated model (with a difference in number of parameters equal to degrees of freedom) (36). In accordance with recommended guidelines (37), within-group effect sizes (Cohen's *d*) from pre-treatment to each major assessment time point were calculated on estimated means from the models for each outcome and raw pooled standard deviations for the relevant assessment period.

Based on the Chi-square difference test, the model with random intercepts and slopes was the best fit to the data for the majority of outcomes. However, due to the small sample size, this model did not converge in the majority of cases. Therefore, for parsimony, we chose to retain a more restricted model with fixed slopes and random intercepts which allowed for different starting values in each outcome. Means and standard deviations for all outcomes across timepoints are found in Table 1 and effect sizes are found in Table 2.

**Table 1:** Estimated means and standard deviations.

	**Pre-treatment M (SD)**	**Mid-treatment M (SD)**	**Post-treatment M (SD)**	**3-month follow-up M (SD)**	**6-month follow-up M (SD)**
PTGI					
Patient	19.40 (10.29)	54.17 (17.53)	64.17 (32.43)	59.20 (34.60)	63.50 (27.36)
Partner	14.60 (15.77)	33.83 (18.28)	55.75 (16.46)	50.00 (22.39)	55.67 (15.32)
SORTS					
Partner	49.58 (8.52)	26.38 (19.37)	21.54 (19.67)	19.08 (16.18)	16.64 (14.84)
QRI					
Support-Patient	3.29 (0.35)	3.50 (0.37)	3.60 (0.42)	3.62 (0.54)	3.62 (0.39)
Support-Partner	3.05 (0.66)	2.86 (0.64)	3.49 (0.42)	3.52 (0.40)	3.29 (0.36)
Conflict-Patient	2.22 (0.48)	2.21 (0.41)	1.87 (0.59)	1.81 (0.49)	1.74 (0.56)
Conflict-Partner	2.37 (0.69)	2.28 (0.69)	2.15 (0.38)	1.96 (0.57)	1.86 (0.40)
Depth-Patient	3.56 (0.27)	3.61 (0.48)	3.60 (0.38)	3.72 (0.33)	3.75 (0.23)
Depth-Partner	3.16 (0.57)	3.28 (0.72)	3.47 (0.52)	3.47 (0.53)	3.42 (0.58)
MSIS					
Patient	118.00 (16.33)	128.67 (15.32)	141.80 (24.80)	141.83 (22.09)	137.50 (18.64)
Partner	128.83 (30.14)	135.33 (29.35)	137.80 (21.97)	146.50 (18.44)	145.33 (19.20)
IPF					
Patient	52.35 (14.48)	47.17 (11.80)	35.94 (12.57)	35.64 (18.14)	34.14 (13.95)
Partner	22.21 (9.10)	25.55 (12.91)	23.54 (7.30)	16.74 (9.24)	18.90 (8.26)
IRI					
Personal Distress-Patient	11.83 (4.07)	10.17 (5.98)	7.00 (6.04)	9.83 (4.17)	10.50 (5.89)
Personal Distress-Partner	11.50 (6.69)	10.33 (4.59)	10.20 (2.95)	9.50 (4.18)	10.00 (3.16)
Fantasy-Patient	13.50 (6.66)	13.00 (6.42)	13.00 (8.34)	10.20 (7.98)	12.83 (8.73)
Fantasy-Partner	11.50 (4.68)	11.33 (4.68)	14.00 (7.31)	11.00 (6.90)	12.17 (6.85)
Perspective Taking-Patient	15.60 (5.81)	17.33 (2.66)	18.00 (2.74)	15.20 (4.21)	18.83 (3.31)
Perspective Taking-Partner	21.00 (3.69)	19.33 (4.97)	21.20 (3.35)	19.67 (3.27)	20.67 (2.16)
Empathic Concern-Patient	15.00 (4.90)	19.83 (4.54)	17.60 (3.05)	18.67 (3.08)	18.67 (3.05)
Empathic Concern-Partner	20.67 (4.89)	20.17 (4.17)	21.60 (3.05)	19.17 (4.58)	20.33 (5.47)

*Raw means and standard deviations are presented. PTGI, Post-traumatic Growth Inventory; QRI, Quality of Relationships Inventory; SORTS, Significant Others Responses to Trauma Scale; CTS-2, Conflict Tactics Scale-2; IPF, Inventory of Psychosocial Functioning; MSIS, Miller Social Intimacy Scale; IRI, Interpersonal Reactivity Index*.

**Table 2:** Cohen's *d* effect sizes.

	**Pre- to mid-treatment**	**Pre- to post-treatment**	**Pre-treatment to 3-month follow-up**	**Pre-treatment to 6-month follow-up**
PTGI				
Patient	2.00	1.44	1.48	1.97
Partner	1.76	2.25	2.04	2.74
SORTS				
Partner	1.01	1.21	1.54	1.78
QRI				
Support-Patient	0.68	0.77	0.71	0.94
Support-Partner	0.47	0.66	0.73	0.81
Conflict-Patient	0.60	0.60	0.73	0.73
Conflict-Partner	0.51	0.63	0.68	0.83
Depth-Patient	0.34	0.49	0.58	0.76
Depth-Partner	0.27	0.38	0.41	0.43
MSIS				
Patient	1.02	0.93	1.10	1.31
Partner	0.41	0.56	0.64	0.68
IPF				
Patient	0.95	1.12	1.01	1.26
Partner	0.24	0.39	0.38	0.43
IRI				
Personal Distress-Patient	0.29	0.35	0.47	0.42
Personal Distress-Partner	0.26	0.35	0.35	0.41
Fantasy-Patient	0.17	0.18	0.20	0.20
Fantasy-Partner	0.05	0.05	0.05	0.06
Perspective Taking-Patient	0.26	0.31	0.30	0.35
Perspective Taking-Partner	0.10	0.15	0.17	0.21
Empathic Concern-Patient	0.58	0.81	0.88	0.95
Empathic Concern-Partner	0.21	0.28	0.26	0.26

*Cohen's d effect sizes calculated using least-squares means estimated from growth models and pooled actual standard deviations for relevant assessment periods. PTGI, Post-traumatic Growth Inventory; QRI, Quality of Relationships Inventory; SORTS, Significant Others Responses to Trauma Scale; CTS-2, Conflict Tactics Scale-2; IPF, Inventory of Psychosocial Functioning; MSIS, Miller Social Intimacy Scale; IRI, Interpersonal Reactivity Index*.

## Results

### Safety Outcomes

#### Relationship Aggression

For patients, there were improvements on minor psychological aggression (*B* = −0.98, *SE* = 0.21, *p* < 0.001, *d* = 1.06–1.56) and no significant change in minor physical assault. The models for minor sexual coercion and minor injury could not be estimated due to infrequent endorsement of these items. Likewise, for partners, the models for minor physical assault, psychological aggression, sexual coercion and injury scales could not be estimated due to low endorsement on these scales. There were no significant changes on emotional or cognitive negotiation strategies for patients (emotional: *d* = 0.18–0.29; cognitive: *d* = 0.03–0.04) or partners (emotional: *d* = 0.16–0.22; cognitive: *d* = 0.22–0.36).

### Outcomes

#### Trauma-Related Outcomes: Post-Traumatic Growth and Behavioral Accommodation

Patients and partners both showed significant improvement in patients' post-traumatic growth (Patient *B* = 7.30, *SE* = 1.56, *d* = 1.44–2.00, Partner *B* = 7.63, *SE* = 1.49, *d* = 1.76–2.74, both *p* < 0.001). In addition, partners showed significant decreases in accommodating behavior over the course of treatment (*B* = −3.86, *SE* = 1.36, *p* < 0.01, *d* = 1.01–1.78).

#### Relationship Quality Outcomes

**Figure 1:**
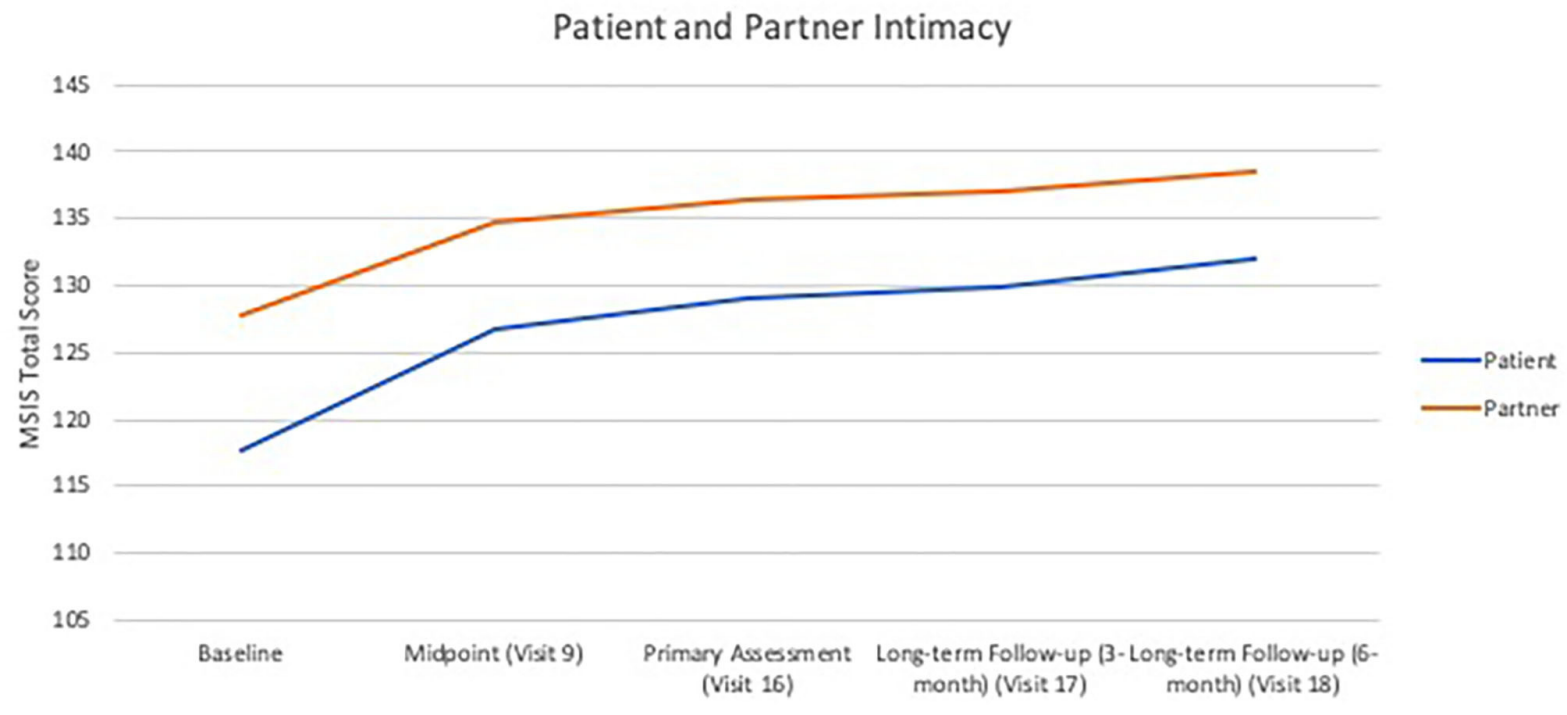
Growth curves of patient- and partner-rated intimacy over treatment visits and follow-ups. Patient-rated intimacy on the Miller Social Intimacy Scale: *B* = 4.12, *SE* = 1.32, *p* < 0.01; Partner-rated intimacy on the Miller Social Intimacy Scale: *B* = 3.10, *SE* = 1.03, *p* < 0.01.

In terms of relationship quality as assessed by the QRI, there was significant improvement in support as rated by both patients and partners (Patients: *B* = 0.06, *SE* = 0.03, *p* = 0.041; *d* = 0.68–0.94, Partners: *B* = 0.08, *SE* = 0.03, *p* = 0.029, *d* = 0.47–0.81). There were no significant improvements in patient-reported conflict (*B* = −0.06, *SE* = 0.03, *p* = 0.062, *d* = 0.60–0.73), but partners reported significant improvement in conflict (*B* = −0.09, *SE* = 0.03, *p* = 0.002, *d* = 0.51–0.83). There were no significant changes found in patient (*B* = 0.03, *SE* = 0.02, *p* = 0.171, *d* =0.34–0.76) or partner-rated (*B* = 0.04, *SE* = 0.03, *p* = 0.093, *d* = 0.27–0.43) depth of relationship. Both patients and partners reported significant increases in intimacy (Patients: *B* = 4.12, *SE* = 1.32, *p* = 0.005, *d* = 0.93–1.31; Partners: *B* = 3.10, *SE* = 1.03, *p* = 0.007, *d* = 0.41–0.68). See Figure 1 for intimacy outcomes.

#### Psychosocial Functioning Outcomes

Finally, patients rated improved overall psychosocial functioning (*B* = −3.20, *SE* = 0.69, *p* < 0.001, *d* = 0.95–1.26), while there was no significant change in partner ratings (*B* = −0.67, *SE* = 0.63, *p* = 0.299, *d* = 0.24–0.43). In terms of empathy as rated by the IRI, patients showed significant improvements (*B* = 0.69, *SE* = 0.32, *p* = 0.037, *d* = 0.58–0.95) in empathic concern but partners did not (*B* = −0.24, *SE* = 0.22, *p* = 0.287, *d* = 0.21–0.26). There were no changes for patients or partners in personal distress (patients: *B* = −0.38, *SE* = 0.24, *p* = 0.126, *d* = 0.29–0.42; partners: *B* = −0.38, *SE* = 0.28, *p* = 0.179, *d* = 0.26–0.41), fantasy (patients: *B* = −0.28, *SE* = 0.47, *p* = 0.456, *d* = 0.17–0.20; partners: *B* = 0.06, *SE* = 0.22, *p* = 0.788, *d* = 0.05–0.06), or perspective taking (patients: *B* = 0.29, *SE* = 0.34, *p* = 0.400, *d* = 0.26–0.35; partners: *B* = −0.11, *SE* = 0.24, *p* = 0.641, *d* = 0.10–0.21).

## Discussion

The couples in this study experienced significant gains in terms of their relational functioning, post-traumatic growth, and behavioral accommodation. They also exhibited gains in or maintenance of strong interpersonal and psychosocial functioning, demonstrating that the combination of CBCT with MDMA for PTSD provides improvements for both partners and the relationship. Additionally, the improvements in minor psychological aggression, and stability of the absence of severe aggression, suggest that the intervention is safe and does not increase a risk of relational or interpersonal harm.

Notably, the improvements in post-traumatic growth were significant for both patients and partners, indicating that both identified growth and change in the partner with PTSD through the course of therapy. Improvements in behavioral accommodation, as assessed by the partner on their own behaviors, demonstrates a greater understanding of the role of accommodation in maintaining PTSD in a relationship, and the choice of the partner to shift their behavior in order to address it.

Improvements in quality of relationship functioning, specifically increases in perception of support and decreases in conflict, demonstrate that this intervention may have promise to strengthen the positive social interactions and diminish the negative social interactions in relationships, both of which are important to recovery post-trauma. Improvements in reported depth of relationship were not significant, which may be partially attributed to the high levels of depth reported by the participants at study baseline. This may speak to couples who are already deeply invested in their relationships as having self-selected into an experimental dyadic treatment for PTSD.

Participants often reported feeling greater connection to others during MDMA-assisted sessions that lasted beyond the therapeutic intervention. Though not formally tested, this experience likely played a role in improvements in intimacy reported in outcome measures. Additionally, improvements in empathic concern and psychosocial functioning for the patient with PTSD suggest a turning toward and engagement with the relationship, and that these results extend beyond the relationship, creating both intra- and interpersonal benefits. This offers a possibility for more holistic improvement and overall well-being. Although partners did not have statistically significant improvement in psychosocial functioning, their baseline scores indicated that they were, as a group, functioning very well, and therefore a large improvement would not have been possible with the intervention. Both partners demonstrated low levels of personal distress related to interpersonal reactivity, potentially accounting for the non-significant findings in this subscale. Patients demonstrated significant improvement in empathic concern, highlighting the relevance of this intervention for improving the well-being of the interpersonal relationship. Partners demonstrated high baseline levels of empathic concern that remained stable over the course of therapy, demonstrating that expressions of empathy can remain stable and improve while engaging in trauma-focused work. Low rates of personal distress and fantasy for both patients and partners were assessed at baseline and remain unchanged.

While the results of the study demonstrate significant improvements, there are numerous limitations to consider. The study sample was very small, and while expected in a proof of concept pilot interventional study, it limits the conclusions that can be drawn. Likewise, the sample was not diverse in terms of ethnicity, race, sexual orientation, and gender identity, suggesting that any conclusions drawn are limited to white, mixed gender, intimate couples. Future studies should place a strong focus on recruiting more diverse and representative samples of participants. The study, by design, was uncontrolled, which means that conclusions regarding the efficacy of the intervention compared to placebo, or either interventional component alone (CBCT or MDMA-assisted psychotherapy), cannot be drawn.

The findings of this pilot study suggest that a larger, controlled study of CBCT + MDMA to explore the relational outcomes of the intervention are warranted. These outcomes also suggest that couple therapy with MDMA may indeed be well-suited for a range of couple-related concerns beyond PTSD, particularly those that are relational in nature. By targeting individual and relational functioning simultaneously, this intervention has the potential to maximize recovery from trauma and enhance present living for those with PTSD and their loved ones.

## Data Availability Statement

The raw data supporting the conclusions of this article will be made available by the authors, without undue reservation.

## Ethics Statement

The studies involving human participants were reviewed and approved by WCGIRB Ryerson University Ethics Board. The patients/participants provided their written informed consent to participate in this study.

## Author Contributions

AW, CM, MM, and AM designed and ran the study. AW drafted the manuscript. RL ran the analyses. All authors reviewed and edited the manuscript.

## Conflict of Interest

CM receives royalties from Guilford Press for the CBCT manual. AW, MM, AM, and CM received salary support funding from MAPS for the study. MM and AM sit on the advisory board for Awakn Life Sciences. The remaining author declares that the research was conducted in the absence of any commercial or financial relationships that could be construed as a potential conflict of interest.
